# Destruction by Fire

**Published:** 1899-12

**Authors:** 


### Editorial.

### DESTRUCTION BY FIRE.

The total destruction of the extensive establishment of the J. B. Lippincott Company involved not only a heavy financial loss, but it is doubtful whether all, or any, of their valuable publications will be saved. The extent of this destruction can only be determined by a thorough examination of their several vaults.

The destruction of this number, prepared for the press, was complete, and required resetting under difficulties. We are under obligations to the editor of the *Dental Cosmos* for his offer of any assistance within his power. We are also indebted to that journal for the half-tone of the Horace Wells bust, the one prepared for this number having been destroyed.

We trust our readers will make due allowance for any deficiencies apparent and for the delay in the issue.

